# CrisprGE: a central hub of CRISPR/Cas-based genome editing

**DOI:** 10.1093/database/bav055

**Published:** 2015-06-27

**Authors:** Karambir Kaur, Himani Tandon, Amit Kumar Gupta, Manoj Kumar

**Affiliations:** Bioinformatics Centre, Institute of Microbial Technology, Council of Scientific and Industrial Research, Sector 39A, Chandigarh 160036, India

## Abstract

CRISPR system is a powerful defense mechanism in bacteria and archaea to provide immunity against viruses. Recently, this process found a new application in intended targeting of the genomes. CRISPR-mediated genome editing is performed by two main components namely single guide RNA and Cas9 protein. Despite the enormous data generated in this area, there is a dearth of high throughput resource. Therefore, we have developed CrisprGE, a central hub of CRISPR/Cas-based genome editing. Presently, this database holds a total of 4680 entries of 223 unique genes from 32 model and other organisms. It encompasses information about the organism, gene, target gene sequences, genetic modification, modifications length, genome editing efficiency, cell line, assay, etc. This depository is developed using the open source LAMP (Linux Apache MYSQL PHP) server. User-friendly browsing, searching facility is integrated for easy data retrieval. It also includes useful tools like BLAST CrisprGE, BLAST NTdb and CRISPR Mapper. Considering potential utilities of CRISPR in the vast area of biology and therapeutics, we foresee this platform as an assistance to accelerate research in the burgeoning field of genome engineering.

**Database URL**: http://crdd.osdd.net/servers/crisprge/.

## Introduction

Genome editing is a method to target any desired sequence in the genome. From past few years, this technique has earned significant achievements in the area of therapeutics or gene therapy with the help of artificially designed nucleases (1). In this method, a sequence-specific DNA-binding domain is fused to a nuclease domain that cuts DNA at intended site with high efficiency but in non-sequence specific manner (2).

The primary tools that are being used to execute genome excision are constructed using zinc fingers (ZF) (3) and transcription activator-like effector (TALE) (4) proteins but they have their own limitations. A new class of nucleases, known as, Clustered regularly interspaced short palindromic repeats/CRISPR-associated proteins (CRISPR/Cas) has emerged in recent times. (5). It is a type of adaptive immunity in bacteria and archaea, which is acquired in response to exposure of foreign genetic material (6). This approach has built a buzz in the scientific community to apply this method in crafting sequence-specific alterations in genomes of various organisms (7).

**Figure 1.**
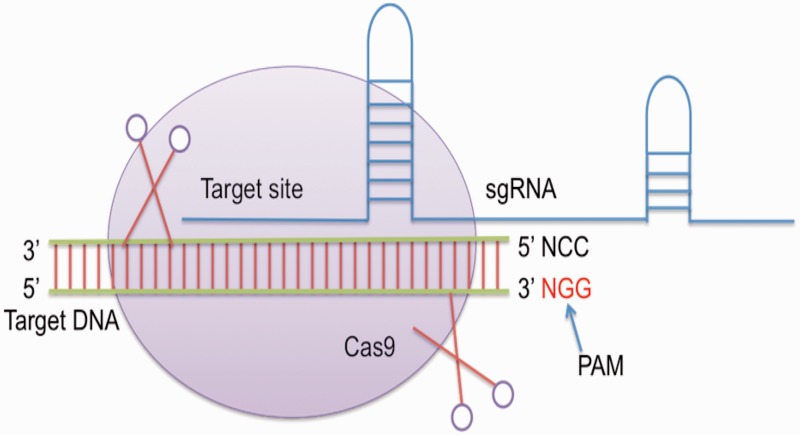
General mechanism of CRISPR/Cas genome editing.

CRISPR was firstly identified in the genome of *Escherichia coli* as uncommon repeat segments (8). Later, it was discovered that CRISPR contain an array of repeat spacer sequences, which are derived from attacking bacteriophages (9). A set of *cas* genes is also present at one end of this array, which are key players in cleaving the foreign genetic material (10). The type II CRISPR/Cas system from bacterium *Streptococcus pyogenes* then emerged as a powerful tool for editing genomes of various organisms (5). It contains a single Cas protein i.e. Cas9 endonuclease and crRNA along with tracrRNA that forms a dual RNA system to cleave a particular target site (11, 12). Single guide RNA (sgRNA) is mainly a chimeric RNA, which is created/generated by merging the 3′-end of crRNA with the 5′-end of tracrRNA. Cas9 requires ‘NGG’ protospacer adjacent motif downstream to the site of target (5) (Figure 1). It has been reported that sgRNA or the chimeric RNA shows more efficiency than using them separately (5).

The breaks induced by Cas9 are repaired by homology directed repair or non-homologous end joining creating alterations i.e. insertions, deletions and substitutions at the target site. CRISPR constructs are easy to design, and plenty of data has been generated in the last few years. The efficiency of this approach motivated Cong *et al*. (11) to execute human genome editing. Subsequently, genome editing using CRISPR was accomplished in model organism namely *Rattus norvegicus*, *Caenorhabditis elegans*, *Danio rerio*, *Mus musculus*, *Drosophila melanogaster*, *Arabidopsis thaliana* and other organisms (12–18).

CRISPR/Cas method has demonstrated wider potential applications comprising knockout (27, 28), knock-in, large chromosomal deletions and replacement of genes in different cells (29–31). This technique has also been successfully utilized to make knockout mice with heritable mutated alleles (32). It is now being used to target long non-coding RNAs *in vivo* (33), to check the changes in proteome after transcription activation (34) and to delete synaptic proteins for studying their functions (35). It is important utility includes correction of genetic disorders like beta thalassemia, and duchenne muscular dystrophy (36–38). This system also helped in creating indels to inactivate human papillomavirus, Hepatitis B virus, HIV-1 and virulent phages (39-43).

In no time, CRISPR/Cas has gained a lot of importance in the field of genome editing. The main aim of CrisprGE is to provide single platform to integrate the growing information being generated by this genome editing approach.

## Materials and Methods

### Data search

Extensive literature search was done, and data were retrieved from PubMed with different combination of keywords comprising ‘Clustered regularly interspaced short palindromic repeats’, ‘CRISPRs’, ‘CRISPR*’, ‘CRISPR’, ‘genome editing’, ‘genome engineering’, etc. The query used for the advanced search option is as follows:

(((((Clustered regularly interspaced short palindromic repeats) OR CRISPRs) OR CRISPR) OR CRISPR*)) AND ((genome editing) OR genome engineering)

With this query, 575 articles were obtained as of April 2015. We extracted articles having data related to organisms and genes, along with the modification generated by this targeting. Reviews and general methodology articles were excluded. Similarly, articles lacking the desired information were also omitted. Finally, **4680** entries were totally extracted.

### Database organization

**Figure 2.**
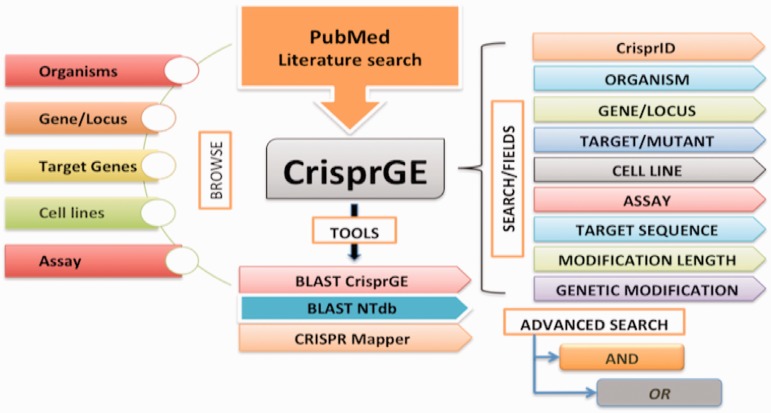
CrisprGE design.

For precise demonstrations, this directory/database is organized to comprehend the different aspects of genome editing (Figure 2) and includes the following fields:

CrisprID: a unique ID is given to each entry.
Organism: all organisms are displayed according to their Latin names (e.g. *Homo sapiens*).
Gene/locus: genes are formatted according to NCBI’s Gene database and literature (e.g. CCR5).
Target sequence: sequence of the target gene from the respective study.
Target/mutant: sequence of the wild-type gene and the modified sequence or mutant.
Cell line: cell lines on which experiments were performed (e.g. HEK293).
Assay: experimental method used to find indels (e.g. sequencing).
Genetic modification: insertion, deletion, point mutation, indels.
Modification length: length of insertion, deletion, indels (e.g. D1, D2).
PMIDs: references are specified as PubMed IDs.

The database is equipped with easy browsing and searching options. Analysis tools like BLAST CrisprGE, BLAST NTdb and CRISPR mapper are also present. Individual entries are hyperlinked to other resources like UniProt, KEGG and PubMed, etc.

### Implementation of web-interface

CrisprGE is constructed using the open source LAMP server on Red Hat Enterprise Linux 5 with MySQL and Apache on the back end. The front end is implemented with PHP. It is freely available at: http://crdd.osdd.net/servers/crisprge/.

## Results

### Database statistics

**Figure 3.**
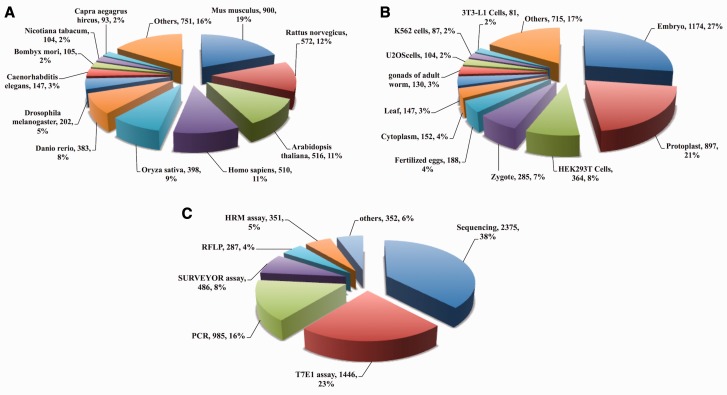
CrisprGE statistics: graphs are representing the statistical distribution of the (**A**) organism (**B**) cell lines (**C**) assay. PCR, polymerase chain reaction; T7E1, T7 endonuclease1 assay; HMA, heteroduplex mobility assay; HRMA, high-resolution melting assay; RFLP, restriction fragment length polymorphism; RE, restriction enzyme assay; CAPS, Cleaved Amplified Polymorphic Sequences; SSA assay, Single-strand annealing assay.

CrisprGE is a dedicated repository having total of 4680 genes edited by CRISPR/Cas approach. It comprises 223 unique genes targeted in 32 model and other organisms along with different modification induced by repair mechanisms. It also contains details of various organisms in which genome editing has been carried out (Figure 3A). The experiments reported in the database have been performed on different cell lines. Out of these, injection of sgRNA constructs in embryo (Figure 3B) is the most commonly applied strategy followed by injection of plant cells and protoplast. There are different methods to detect indels at the target site. Amongst them, most widely used method in the literature was that of sequencing, followed by T7 Endonuclease I assay (Figure 3C).

**Figure 4.**
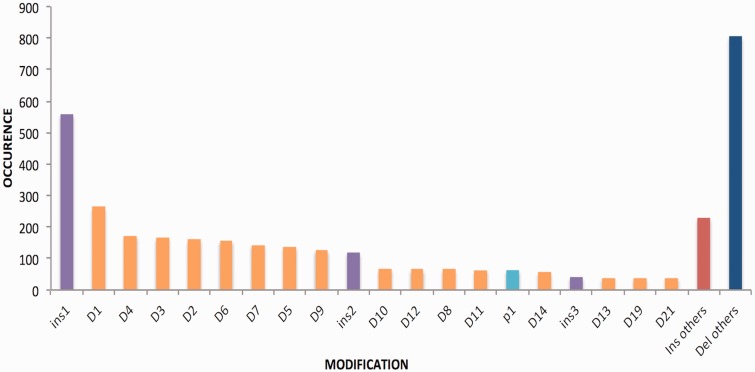
Bar graph is signifying length of insertions and deletion of various genes. Del, deletion; Ins, insertion and p, point mutation.

The modifications achieved on the target sites are mainly insertions or deletions, point mutations and in some cases both. The range of deletions has been observed between 1 and 294 24 bp and that of insertion from 1 to 1837 bp. It has been seen that most of the deletions and insertions created were of 1 bp followed by 3 bp or 4 bp. The deletion pattern is shown in Figure 4.

In this depository, we have also incorporated top 20 genes Table 1, which are targeted at least 70 times by CRISPR/cas method. Among them, Tyr and alcohol dehydrogenase 1 (ADH1) are the most commonly edited genes, followed by phytoene desaturase (PDS), Prkdc and Tet1 from *M. musculus* and TT4 from *A. thaliana*. List of all genes and organism wise frequency distribution are also provided (see Supplementary Tables S1 and S2, respectively).

**Table 1.** List of top genes targeted by CRISPR/Cas system

**Genes**	Number of entries	Organism
**Tyr**	252	Mus musculus, Rattus norvegicus, Xenopus tropicalis, Danio rerio
**ADH1**	238	Arabidopsis thaliana, Nicotiana benthamiana
**PDS**	155	Nicotiana tabacum, Nicotiana benthamiana, Oryza sativa, citrus sinensis
**Prkdc**	125	Rattus norvegicus, Mus musculus
**Tet1**	118	Rattus norvegicus, Mus musculus
**TT4**	108	Arabidopsis thaliana
**B2m**	95	Rattus norvegicus, Mus musculus
**YSA**	92	Oryza sativa
**Tet2**	88	Rattus norvegicus, Mus musculus
**DDM1**	87	Glycine max
**CCR5**	86	Homo sapiens
**PCSK9**	81	Mus musculus
**DMD**	80	Homo sapiens
**fh**	72	Danio rerio
**Pcdh**	72	Homo sapiens
**HBB**	70	Homo sapiens
**ApoE**	69	Rattus norvegicus, Danio rerio
**Tet3**	68	Rattus norvegicus, Mus musculus
**Prf1**	67	Rattus norvegicus, Mus musculus
**PDS3**	66	Arabidopsis thaliana, Nicotiana benthamiana

DMD, duchenne muscular dystrophy.

## 

### Data retrieval

#### CrisprGE browse

CrisprGE has been provided with easy browsing options. Users can browse it by any of the five fields namely, Organism name, Gene/Locus, Target sequence, Cell line and Assay see (see Supplementary Figure S1).

### Database search and advanced search

In basic search option, user can enter query in the box and can search for provided fields. Search output has information on essential components like CrisprID, organism, gene, target, modification, location and PMIDs (see Supplementary Figure S2). Sorting and filtering functionality is also offered in the search output.

Along with the simple search, a user-friendly advanced search tool is also offered for extensive data search. User can apply logical operators (=/like) along with conditional operators (AND/OR) on various fields such as organism, gene, target and modification, etc. User can add ‘N’ number of keywords just by clicking on Add button and can build final query (see Supplementary Figure S3). The output gives information, which can be sorted, and further filtered based on specific keywords using a filter box. Additionally, hints on allowed search keywords are also provided to assist users.

### Analysis tools

Various tools have been assimilated to assist analysis of CRISPRs. ‘BLAST NTdb’ tool is available in CrisprGE to support users to align their target sequence against the NCBI non-redundant nucleotide database. It was built by downloading standalone BLAST programs from NCBI BLAST ftp (ftp://ftp.ncbi.nlm.nih.gov/blast/db/) site. After installation, this is implemented on the Red Hat Enterprise Linux 5 web server. A text box is given in which query sequence can be inserted in Fasta format. Default parameters such as Expected value (10), Scoring Matrix (BLOSSUM62), Alignment view (Pairwise), etc. are used to query target sequence. The output displays alignment, graphical view and score. ‘BLAST CrisprGE’ tool will help user to align their desired sequence with the target sequences from CrisprGE repository. It helps user to find best possible target site hits for their gene. Default parameters and the resulting output of this tool are similar to nucleotide BLAST output.

‘CRISPR Mapper’ can be utilized to find possible off-target sequence regions within particular gene or genome. It helps user to explore the perfectly matching target sequences on user provided nucleotide sequence, which generates a list of target sites with details. Output of this tool displays the CrisprID, organism name, gene or locus, target sequence, start position along with the associated genetic modification and its length (see Supplementary Figure S4).

Each entry in this databank is curated manually and further verified by cross-checking. The tools included in web server are also checked for proper working. It would be updated half yearly/yearly to encompass newer records.

### Comparison of genome editing methods

Besides CRISPR/Cas, artificially designed nucleases like ZF proteins and TALEs are also exploited for genome editing (19, 20). Both these nucleases have a DNA binding and catalytic domain (21, 22). The catalytic domain in ZFNs and TALENs is derived from FokI (type II restriction endonuclease) while in CRISPR system it originates from Cas9 nuclease. Although, ZFNs and TALENs have been successfully used for genome editing, they have some restraints, specifically on their delivery, due to large size (23) and may also have toxicity (24). Further, there is always a need to reconstruct new enzyme for every new DNA target. In CRISPR/Cas, a single Cas9 nuclease is sufficient to perform these tasks (25).

We compared the effectiveness and frequency of excision mediated by all three approaches of genome editing. The genes targeted by CRISPR/Cas of our resource were checked in EENdb- a database of ZFNs and TALENs-based genome editing (26). List of genes targeted by all these methods is shown in Table 2. For example, CRISPR/Cas-mediated editing of human CCR5 gene has been 76.00% efficient whereas ZFNs and TALENs achieved efficiency of 16.70% and 20.00%, respectively. CRISPR/Cas-based editing of ben-1 gene in *C. elegans* was 88.00% efficient followed by 3.50% using other two techniques. However, in few cases, the other two techniques have slightly better efficiency e.g. gene ADH1 of *A. thaliana*. These observations suggest that CRISPR/Cas is comparatively more efficient than other methods of genome editing.

**Table 2.** Comparison of genome editing efficiency with different methods

Organism/species	Gene	Method	Modification method	Efficiency (%)	Efficiency detection method	PMID
**Human (*Homo sapiens*)**	CCR5	CRISPR/Cas9	NHEJ	76	T7E1 assay/ Sequencing	23939622
		ZFNs	NHEJ	16.70	MDNA/SSA assay	19470664
		TALENs	NHEJ	20	MDNA	21179091
**Human (*Homo sapiens*)**	HBB	CRISPR/Cas9	NHEJ	70	T7E1 assay/ Sequencing	23939622
		ZFNs	NHEJ, HR	2.1/12.9	Sequencing	21898685
		TALENs	NHEJ	NA	Reporter gene addition assay	22301904
**Rat (*Rattus norvegicus*)**	Prkdc	CRISPR/Cas9	NHEJ	66.70	T7E1 assay	24598943
		ZFNs	NHEJ	NA	Sequencing	22981234
		TALENs	NA	NA	NA	NA
**Worm (*Caenorhabditis elegans*)**	ben-1	CRISPR/Cas9	NHEJ	88	Sequencing	24013562
		ZFNs	NHEJ	3.50	MDNA & high-throughput sequencing	21700836
		TALENs	NHEJ	3.50	MDNA	21700836
**Zebrafish (*Danio rerio*)**	gria3a	CRISPR/Cas9	NHEJ	61	T7E1 assay	23360964
		ZFNs	NHEJ	26	Sequencing	21822241
		TALENs	NHEJ	15	Sequencing	21822241
		TALENs	NA	NA	SSA assay	21493687
**Thale cress (*Arabidopsis thaliana*)**	ADH1	CRISPR/Cas9	NHEJ	8	HRMA, sequencing	24836556
		ZFNs	NHEJ	16	Restriction-enzyme- resistance assay	20508152
		TALENs	NHEJ, HR	NA	SSA assay, & restriction-enzyme- resistance assay	21493687
**Silk worm (*Bombyx mori*)**	BLOS2	CRISPR/Cas9	NHEJ	35.60	PCR	24165890
		ZFNs	NHEJ	0	Reporter gene disruption assay/ direct sequencing	20692340
		TALENs	NHEJ	0.45	Reporter gene disruption assay	23028749

NHEJ, non homologous end joining; HR, homologous recombination; PCR, polymerase chain reaction; ZFNs, zinc finger nucleases; TALENs, transcription activator like effector nucleases; T7E1, T7 endonuclease1 assay; HRMA, high resolution melting assay; SSA, single strand annealing; MDNA, mismatch-detection nuclease assay.

## Discussion

CRISPR/Cas-based genome editing has been extensively explored since invention of sgRNA. This method was successfully applied for excising genome of various organisms namely humans (11, 44), *M. musculus* (45), *D. rerio* (46), *A. thaliana* (18), etc. These findings lead to the generation of a huge amount of data on genome editing. CrisprGE is the first specialized resource to encompass vital data on CRISPR/Cas-based genome editing. Presently, it comprises a total of 4680 entries of 223 unique genes from 32 model and important organisms. Prior to our resource, only 439 entries of TALEN and 340 of ZFN-mediated genome editing were available in EENdb (15). Also in EENdb they have provided only eight data fields while CrisprGE covers 12 data fields each offering significant information.

We have analysed the pattern of modifications mediated by CRISPR/Cas method. We observed that each kind of mutations like insertions, deletions and point mutations have been carried out using this method. Deletions and insertions range from as small as 1 bp to as large as several kilo base pairs. However, efficiency of small indels like 1–2 bp was high in different organisms but large indels have also been also performed with good efficiency (47, 48). Although this technique has been majorly applied to target a particular location in genome. Lately, it has also exhibited potential to target many genes or even various locations within a gene simultaneously with high efficiency. For example, *Tet1*, *Tet2* and *Tet3* genes were aimed in *M. musculus* (49), multiple locations in *Coe* gene of *Ciona intestinalis* (50) as well as *w* gene of *D. melanogaster* (47).

We have provided a user-friendly web server with data retrieval capabilities. Standard browse, search, and advanced search options are offered for easy access to data. Advanced search facility help users to explore multiple terms and restrict the search in one click. Sorting and filtering options help users to refine their search further. ‘How to use’ section with step-by-step pictorial representation is offered on web server. In addition, various analysis tools have also been integrated for further help, e.g. Using KEGG Mapper analysis tool, we found those targets genes were involved in various metabolic pathways. We have checked that, genes, which are frequently targeted e.g. Tyr (tyrosinase) is involved in Tyrosine metabolism; ADH1 is involved in glucose metabolism and PDS is engaged in Carotenoid biosynthesis. Thus, this suggests that CrisprGE harbor genes, which regulate various biological pathways.

The only limitation here is that data on genome editing is increasing very fast as evident from recent literature; therefore, it is necessary to keep the database up to date. Each record in CrisprGE is curated manually at the time of data extraction and further cross-checked. The same strategy would be continued for addition of new entries preferably on half-yearly/yearly basis. Further emphasis would be given on to incorporate newer analysis tools for CRISPR.

Genome editing has generated a large amount of data so there is an irresistible need to develop a storehouse that can accommodate high throughput data. In a very short span, this method has successfully been applied to knock in and knock out genes, creating mutations and also large chromosomal deletions. It has also shown therapeutic potential in curing genetic disorders and inhibiting viral infections, etc. Therefore, we expect that CrisprGE resource would assist the wider scientific community working on different aspects of CRISPR-based genome editing.

## Supplementary Material

Supplementary Data
