# Supplementary material for: CrisprGE: a central hub of CRISPR/Cas-based genome editing
Source: Database (Oxford). 2015 Jun 27;2015:bav055. doi: 10.1093/database/bav055 (PMC4483309; doi:10.1093/database/bav055)
Supplement: Supplementary Data [file supp_2015_bav055_index.html]

CrisprGE: a central hub of CRISPR/Cas-based genome editing — Supplementary Data 

# CrisprGE: a central hub of CRISPR/Cas-based genome editing

## Supplementary Data

files

- Supplementary Data - zip file
