# Supplementary material for: CrisprGE: a central hub of CRISPR/Cas-based genome editing
Source: Database (Oxford). 2015 Jun 27;2015:bav055. doi: 10.1093/database/bav055 (PMC4483309; doi:10.1093/database/bav055)
Supplement: Supplementary Data [file supp_bav055_suppl_data.zip › supplementary Data1.doc]

**CrisprGE: A central hub of CRISPR/Cas based genome editing**

Karambir Kaur, Himani Tandon, Amit Kumar Gupta and Manoj Kumar*

Bioinformatics Centre, Institute of Microbial Technology, Council of Scientific and Industrial Research, Sector 39A, Chandigarh-160036, India

* To whom correspondence should be addressed: [manojk@imtech.res.in](mailto:manojk@imtech.res.in)

**Figure S1**. Screenshot representing CrisprGE Browse

**Figure S2**. Schematic representation of search option with top displaying search form, output and bottom demonstrating detailed information of an individual entry

**Figure S3**. Screenshot depicting advanced search at the top and subsequent output at the bottom.

**Figure S4**. Screenshot illustrating Crispr mapper analysis tool with output.
